# The Targeted Transduction of MMP-Overexpressing Tumor Cells by ACPP-HPMA Copolymer-Coated Adenovirus Conjugates

**DOI:** 10.1371/journal.pone.0100670

**Published:** 2014-07-07

**Authors:** Shuhua Li, Juanzhi Chen, Huiyong Xu, Jie Long, Xiaobin Xie, Yajie Zhang

**Affiliations:** 1 Department of Pathology and Stomatology, School of Basic Medical Sciences, Southern Medical University, Guangzhou, People's Republic of China; 2 Department of Pathology, School of Basic Medical Sciences, Guangzhou Medical University, Guangzhou, People's Republic of China; 3 Department of Pathology and Stomatology, Nanfang Hospital, Southern Medical University, Guangzhou, People's Republic of China; Stanford University, United States of America

## Abstract

We have designed and tested a new way to selectively deliver HPMA polymer-coated adenovirus type 5 (Ad5) particles into matrix metalloproteinase (MMP)-overexpressing tumor cells. An activatable cell penetrating peptide (ACPP) was designed and attached to the reactive 4-nitrophenoxy groups of HPMA polymers by the C-terminal amino acid (asparagine, N). ACPPs are activatable cell penetrating peptides (CPPs) with a linker between polycationic and polyanionic domains, and MMP-mediated cleavage releases the CPP portion and its attached cargo to enable cell entry. Our data indicate that the transport of these HPMA polymer conjugates by a single ACPP molecule to the cytoplasm occurs via a nonendocytotic and concentration-independent process. The uptake was observed to finish within 20 minutes by inverted fluorescence microscopy. In contrast, HPMA polymer-coated Ad5 without ACPPs was internalized solely by endocytosis. The optimal formulation was not affected by the presence of Ad5 neutralizing antibodies during transduction, and ACPP/polymer-coated Ad5 also retained high targeting capability to several MMP-overexpressing tumor cell types. For the first time, ACPP-mediated cytoplasmic delivery of polymer-bound Ad5 to MMP-overexpressing tumor cells was demonstrated. These findings are significant, as they demonstrate the use of a polymer-based system for the targeted delivery into MMP-overexpressing solid tumors and highlight how to overcome major cellular obstacles to achieve intracellular macromolecular delivery.

## Introduction

Adenovirus (AdV) is a widely used vector for cancer gene therapy because of its capacity for transgene expression in both dividing and nondividing cells [1]–[4]. However, when they are to be delivered intravenously to treat primary tumor or metastatic disease, the wide tissue distribution of the coxsackie and adenovirus receptor (CAR, the primary receptor for adenovirus type 5) precludes target selectivity, and neutralization of adenovirus by pre-existing antibodies can ablate the delivery. Further, the virus can provoke immune responses which prevents repeated dosing and limits the duration of therapeutic gene expression [5], [6]. These factors have largely limited therapeutic use of adenovirus to local or direct administration. A useful viral gene therapy vector should be protected from neutralizing antibodies and enable delivery to target cells.

Addressing these challenges necessitates alternative approaches to redirect AdV type 5 (Ad5) for CAR-independent cellular internalization. Synthetic materials such as cationic lipids and cationic polymers have been used to modify AdV to improve cellular uptake [7]–[14]. However, pEGylated virus still exhibits CAR-mediated infection, which results in nonspecific membrane activity [15]–[17]. Thus, hydrophilic poly-[N-(2-hydroxypropyl) methacrylamide] (pHPMA) has received attention as a more attractive polymer for this purpose. A previous study showed that pHPMA is the optimal means of modification, as it ablates normal pathways of Ad5 infection completely, prolongs the circulation time in blood [18], increases the accumulation of Ad5 by the EPR (enhanced permeability and retention) effect [19] and permits incorporation of a range of targeting molecules and biological effectors to enhance properties such as programming tropism, as well as tissue penetration [20]–[30]. To the best of our knowledge, a detailed investigation of pHPMA modification used to potentiate Ad5 infection and target solid tumor cells has not been reported.

We report here the development of a covalent coating and retargeting strategy using a multivalent hydrophilic polymer based on poly-[N-(2- hydroxypropyl) methacrylamide] (pHPMA) and activatable cell-penetrating peptides (ACPPs). Cell-penetrating peptides (CPPs) are peptides that can translocate through the cellular membranes, such as TAT, Antp, VP22, and polyarginine [31]–[35], and are being used to deliver various cargoes into the cell, including proteins, DNA, antibodies, toxins and nanoparticulate drug carriers (including pHPMA) [36]–[44]. ACPPs are polycationic peptides (polyarginine) which are neutralized by polyanionic (polyglutamic acid) sequences through the fusion of cleavable linkers. Only in the immediate vicinity of extracellular proteases [matrix metalloproteinases (MMPs)] in tumors are ACPPs released from the polycationic peptides, allowing their cargo to attach to and enter cells. Because MMP-2 and MMP-9 are proteases mostly overexpressed by tumors, they were chosen as the initial primary targets [45]–[52]. Incorporation of targeting ACPPs onto the polymer-coated virus enables CPP-mediated and CAR-independent binding and uptake into cells overexpressing MMPs. Thus, ACPPs are an effective means of altering viral tropism and targeting tumor cells. Based on the selective local targeting and activation of CPPs, multivalent polymeric modification of adenovirus may provide the ability to retarget viruses to infect human carcinoma cells. As a non-genetic process, the technology is simple, versatile and should yield viruses with an improved safety profile.

## Materials and Methods

### Chemicals

5-or6-(N-Succini- midyloxycarbonyl)-3',6'-O,O'-diacetylfluorescein (CFSE) and propidium iodide (PI) were purchased from Nantong pharmaceutical Co., Ltd. (Jiangsu, China). The ACPP (EEEEEEEE-PLGLAG-RRRRRRRRN) was synthesized based on our design by Invitrogen Co., Ltd. (Shanghai, China). All other chemicals and reagents were purchased from Sigma Chemical Co. (St. Louis, MO, USA) and used as indicated.

### Synthesis of HPMA copolymer

The monomers of HPMA, methacryloyl-glycyl-glycine p-nitrophenyl ester (MAGGONp), were synthesized according to previously published procedures [23], [24]. Copolymers (pHPMA-ONp) containing N-(2-hydroxy-propyl) methacryl amide (90 mol%) and methacryloyl-Gly-Gly-4-nitrophenoxy ester (10 mol%) with average molecular weight of 16500 were prepared as follows: a polymeric precursor containing reactive ONp estergroups (P') was synthesized by free radical precipitation copolymerization of the monomers of HPMA and MAGGONp in an acetone/DMSO mixture at 50°C for 24 h with 2,20-azobisisobutyronitrile (AIBN).

### Cells and viruses

A549 human lung carcinoma cells and MDA-MB-231 human breast adenocarcinoma cells were grown in Dulbecco's modified Eagle's medium (DMEM) supplemented with 10% fetal calf serum (FCS) and 2 mM glutamine. Human bronchial epithelial (HBE) cells and HepG2 human hepatocarcinoma cells were grown in medium RRPMI-1640 supplemented with 10% fetal calf serum (FCS). The AdMax system was employed to generate adenovirus vectors. Recombinant Ad5 viruses with E1 and E3 deleted and expressing enhanced green fluorescent protein (eGFP) under the direction of the mCMV promoter, termed Ad-eGFP, were purified on CsCl gradients, and the viral titers were determined by a plaque assay and the titer was adjusted to 1.2×10^12^ plaque-forming units/ml.

### RNA Preparation and RTFQ-PCR

Total RNA was extracted from human lung carcinoma cells A549,human breast adenocarcinoma cells MDA-MB-231, Human bronchial epithelial cells HBE and human hepatocarcinoma cells HepG2 with Trizol reagent (Invitrogen, USA) according to the manufacturer's instructions. The quantity of the RNA in the extraction was determined by measuring the absorbance ratio of A260 and A280 using spectrophotometer (Hitachi, Japan).For cDNA synthesis, 3.0 µg of total RNA was processed directly to cDNA by reverse transcription in 20 µL Prime Script RT Enzyme MixI (TAKARA, JPN) according to the manufacturer's protocol.

To quantify the RAGE mRNA expression in A549, MDA-MB-231, HBE, and HepG2, we performed RTFQ-PCR on a LightCycler (Bio-Rad CFX96, USA) using the SYBR Green method. Primers for MMP2 were designed to amplify 180 bp exon of genomic DNA (NM-004530: forward 5′- TGG CAA GTA CGG CTT CTG TC -3′, reverse 5′- TTC TTG TCG CGG TCG TAG TC-3′); Primers for MMP9 were designed to amplify 224 bp exon of genomic DNA (NM-004994: forward 5′- TGG GGG GCA ACT CGG C -3′, reverse 5′- GGAATGATCTAAGCCCAG -3′); Primers for GAPDH were designed to amplify 223 bp exon of genomic DNA (NM-002046: forward 5′- GAA GGT CGG AGT CAA CGG AT -3′, reverse 5′- CTG GAA GAT GGT GAT GGG AT -3′). RTFQ-PCR assay was performed in 50 µL reaction mixture with 25 µL SuperReal PreMix Plus, 10 µmol/L Forward primer, 1.5 µL reverse primer, 3 µL cDNA and 19 µL RNase-free ddH_2_O. The thermal cycles were: 1 cycle of 95°C for 15 min, followed by 40 cycles (for MMP9)or 35 cycles(for MMP2) at 95°C for 10 s, 60°C for 10 s, 72°C for 10 s for measuring fluorescence signals. In order to confirm the specificity of PCR products, inspection of agarose gel electrophoresis and the Alpha innotech softs were used. The mRNA expression level was presented as an OD ratio of MMP2 and MMP9 to that of GAPDH.

### Protein isolation and measurement with western blot

For protein extraction, 10^6^ cells of A549, MDA-MB-231, HBE and HepG2 were homogenized in lysis buffer containing 50 mM KCl, 250 mM cane sugar, 20 mM Tris–Cl, 100 mM NaCl, and 1 mM PMSF. The Protein Analyzer (Pharmacia Biotech, Piscataway,NJ, USA)was used to determine the total protein concentration. Each sample was loaded on an sodium dodecyl sulfate–poly -acrylamide electrophoresis gel and blotted with affinity-purified polyclonal antibodies specific for MMP-2 and MMP-9 (Santa CruzBiotech- nology, CA, USA),in 5% non-fat dry milk in Tris-buffered saline containing 0.1% Tween 20. After washed with PBS, the membranes were incubated with the corresponding second antibody. The resulted membranes were visualized using the LumiGLO Chemiluminescent Substrate solution (Cell Signaling,Danvers, MA, USA). The ratio of MMP-2 and MMP-9 to β-actin was used to evaluate protein mass as a function of SDS treatment.

### Immunofluorescence

A549, MDA-MB-231, HepG2 and HBE cells were grown on coverslips and co-cultured with or without doxycycline (10 mg/L). After 48 h, cells were washed 3 times with cold PBS, followed with4% paraformaldehyde at 40′. The resulted cells were permeabilized in 0.1% Triton X-100 for 10 minutes before blocked in 5% bovine serum albumin in PBS for 30 minutes. Then the coverslips were stained with primary antibodies for MMP-2 (Santa CruzBiotech- nology, 1∶100), followed with Alexa 488 conjugated anti-rabbit secondary antibodies (Invitrogen, 1∶400). DAPI(Sigma)was used for nuclear counterstaining, while images were captured using an Nikon camera (LH-M100CB-1, Nikon, JP).

### Effects of ACPP analysis

To determine the effects of MMPs on the activity of ACPP, A549, MDA-MB- 231,HBE and HepG2 cells (5×10^5^) were aliquoted into 6-well plates after cultured at 37°C for 24 hours and treated with/without doxycycline at 10 µg/ml for 48 h. ACPP with a final concentrations of 200 umol/L was added to infect the cells for 4 hours. The media was then discarded, and the cells were washed with phosphate balanced solution (PBS, pH 7.0) six times. The FITC fluorescence images were recorded with a Nikon camera (LH-M100CB-1, Nikon, JP).

### Virus modification

A random copolymer (pHPMA-ONp) was prepared and the coating of Ad5 particles was performed by adding 25 µL pHPMA-ONp (10 mg/ml in H_2_O) to 10^10^ Ad-eGFP particles in 100 ml 10% glycerol/PBS (pH 7.8), and incubated at 4°C for 12 h to form polymer-coated adenovirus (pc-Ad-eGFP). For the linkage of ACPPs, following an initial 2 h incubation of virus with pHPMA-ONp, ACPPs were added to a final concentration of 250 µg/ml and incubated for an additional 10 h to form ACPP-pc-Ad-eGFP. In characterization studies, the modified virus was purified from free polymer and free targeting agents using S400 spin columns 27-5140-01 (GE Healthcare).

### Sizing of conjugates by dynamic light scattering (DLS)

Particle size after virus modification was measured by dynamic light scattering (DLS). Ad-eGFP (ZetaSizer, Malvern, UK) conjugates pc-Ad-eGFP and ACPP-pc-Ad-eGFP (1×10^10^ Ad5, 50 µl double distilled H_2_O) were mixed with 950 µl double distilled H_2_O. Particle sizing measurements were performed at a wavelength of 659.0 nm with a detection angle of 90° at RT.

### Uptake and infectivity assays

Cells (10^4^) were aliquoted into 96-well plates and infected 24 h later with 10^4^ particles per cell of virus or modified virus in 200 µl DMEM with 10% FCS. Green fluorescent protein (GFP) expression was assessed by microscopy. A Nikon TI-S microscope was employed and images were recorded with a Nikon camera (LH-M100CB-1, Nikon, JP).

For quantitation of GFP fluorescence, the cells were lysed in 100 ml Triton X- 100 (0.2% v/v in 100 mM potassium phosphate, pH 7.8) in 96-well plates following the infection with Ad-eGFP and the conjugates for 48 h. GFP fluorescence (λ_ex_ 488 nm and λ_em_ 538 nm) was measured with a Fluoroskan plate reader (Multiskan GO, Thermo Scientific) and expressed as relative fluorescence units (RFU).

FACS analysis was used to monitor infection of cells by the virus. A549 cells, at a concentration of (2×10^5^ cells)/(2 ml DMEM/10% FCS), were incubated in 6-well plates at 37°C and allowed reach 90% confluence before the addition of 10^9^ particles of Ad-eGFP, pc-Ad-eGFP or ACPP-pc-Ad-eGFP labeled with PI. Cells were trypsinized, centrifuged (2 min, 1500 g) and washed in PBS 48 h later. Association of PI-labeled virus with cells was measured using a FACS calibur flow cytometer with an argon laser (λ_ex_ 540 nm and λ_em_ 625 nm).

For virus neutralization assays, human serum containing Ad5 neutralizing antibodies (NAb) was diluted 1∶20 in PBS and heated to 56°C for 20 min to inactivate complement. Diluted serum (100 µL) was then incubated with Ad-eGFP and ACPP-pc-Ad-eGFP at 37°C for 20 min before the culture medium was diluted to 10^8^ particles/100 µL. Cells were washed once with PBS before virus solutions were added. After 48 h incubation, the A549 cells were plated in 96-well plates (10^4^ cells per well) and the GFP expression of these samples was measured. The results expressed as the percentage of the signal obtained in the absence of human serum.

### Retargeting polymer-coated adenovirus to alternative cancer cells

HBE, A549, MDA-MB-231 and HepG2 cells were seeded into 96-well plates (10^4^ cells per well) and infected 24 h later with 10^4^ particles per cell of retargeted virus in 200 µL DMEM supplemented with 10% FCS (A549, MDA-MB-231) or 200 µL RRPMI-1640 medium supplemented with 10% FCS (HBE, HepG2). For quantitation, the cells were lysed in 100 µL Triton X-100 (0.2% v/v in 100 mM potassium phosphate pH 7.8) and GFP fluorescence (λ_ex_ 488 nm and λ_em_ 538 nm) was measured with a Fluoroskan plate reader (Multiskan GO, Thermo Scientific) and expressed as relative fluorescence units (RFU).

### Intracellular distribution of conjugates

Cells plated on sterile coverslips in 6-well plates (2×10^5^ cells per well) were grown for 24 h to reach 90% confluence. Cells were incubated with the ACPP-pc-Ad-eGFP conjugate or with pc-Ad-eGFP (which lacks the ACPP) for 1 h at 37°C. For localizations studies involving specific organelle markers(5- or6-(N-Succini- midyloxycarbonyl)-3',6'-O,O'-diacetylfluorescein,CFSE) and propidium iodide (PI) were employed. For studies of internalization, experiments included the incubation of cells with double-labeled conjugates: (FITC)ACPP-pc-Ad-eGFP(PI) or with pc-Ad-eGFP (PI). For the time-dependence studies, cells were incubated with ACPP-pc-Ad-eGFP (PI) or pc-Ad-eGFP(PI) for 20 min, 2 hr or 4 h. After all incubations, cells were washed thoroughly with PBS. The cells were visualized with a Nikon TI-S microscope and images recorded with a Nikon camera (LH-M100CB-1, Nikon, JP)

## Results

**Figure 1:**
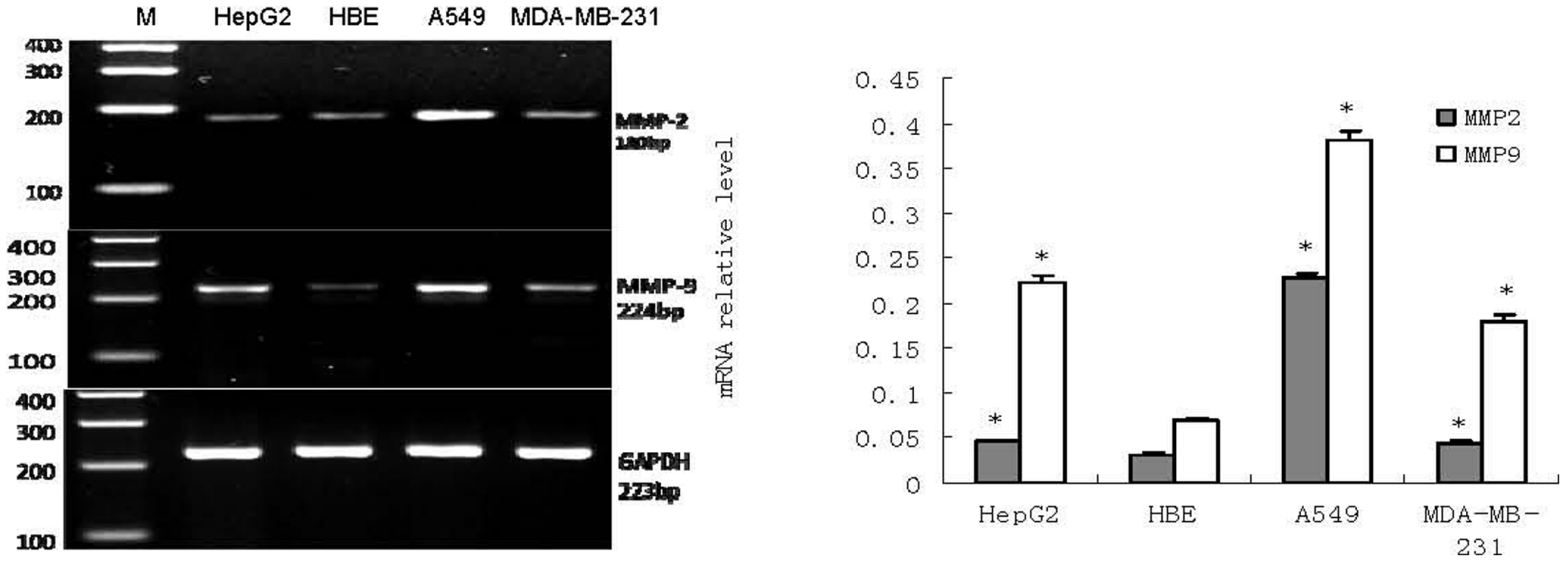
mRNA expression of MMP-2 and MMP-9 in HepG2, HBE, A549 and MDA-MB-231 (n = 3). RTFQ-PCR analysis *Left*, a representative RTFQ-PCR; *Right*, shows quantitative data of MMP-2 and MMP-9 mRNA level, GAPDH was used as an internal control. Data are the means ± SEM. *P<0.05 compared with the HBE cell.

**Figure 2:**
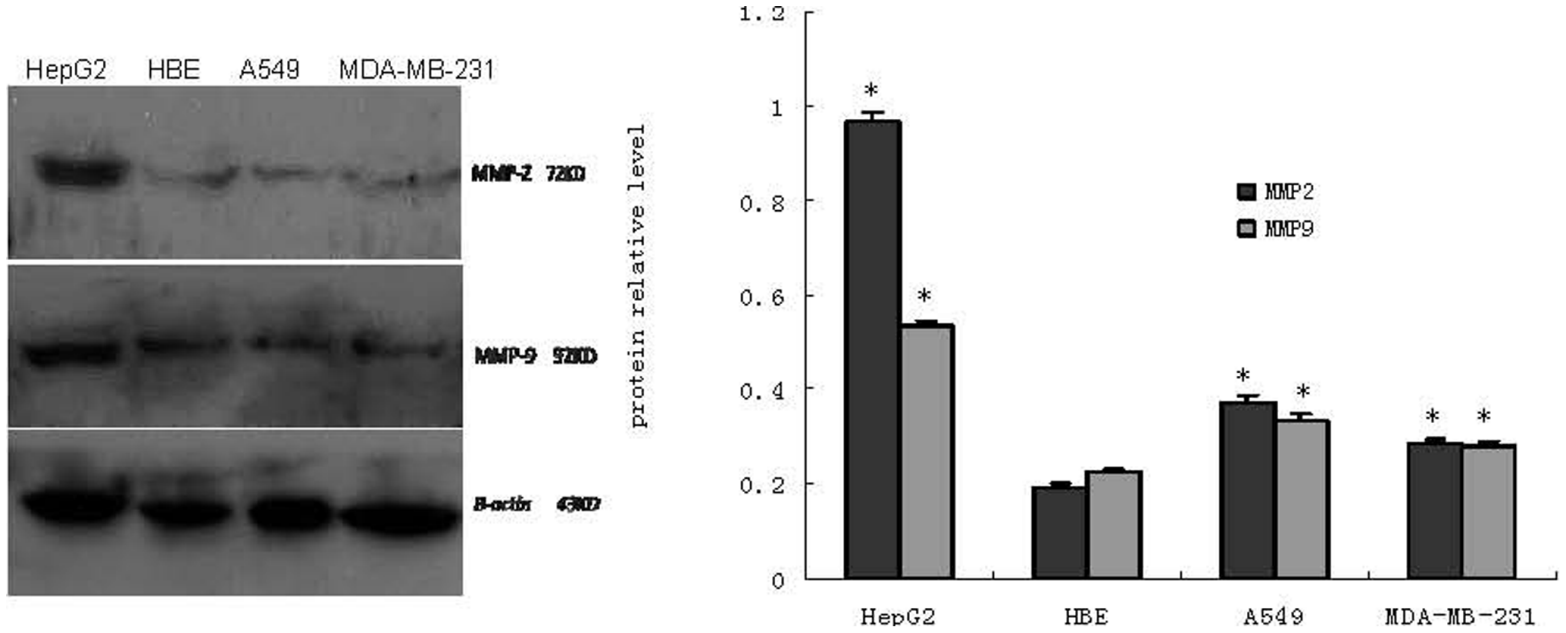
Protein of MMP-2 and MMP-9 in cell lines HepG2, HBE, A549 and MDA-MB-231 (n = 3). *Left*, a representative Western blot; *Right*, densitometric analysis of the representative Western blot, *bars* represent the relative amounts of MMP-2 and MMP-9. Data are the means ± SEM. *P<0.05 compared with the HBE cell.

mRNA and protein expression levels of MMP2and MMP9 were examined in cultured cell lines from A549, MDA-MB-231, HBE and HepG2. Unlike in HBE cell lines, both MMP2 mRNA and protein were overexpressed in these three cancer cell lines as indicated by the observed PCR products at the expected size of 180 bp (Figure 1) and the specific protein binding band at the expected size of 72 kDa (Figure 2). also In addition, RTFQ-PCR and western blot analysis revealed that the levels of both MMP9 mRNA and protein in the three cancer cell lines were higher than in HBE cell line, as indicated by the PCR products at the size of 224 bp (Figure 1) and the specific protein binding band at the size of 92 kDa (Figure 2). These results indicated the expressions of MMP2 and MMP9 were different in these 4 cell lines: overexpressed in cancer cells while under- expressed in HBE cell line for both mRNA and protein.

MMPs are the best characterized soluble proteases in the overexpressed tumors and are responsible for the degradation of extracellular matrix. We integrated substrates(-PLGLAG-) as cleavable linkers between the polyanion inhibition (EEEEEEEE) and polycation sequence(RRRRRRRRN), so that the polycation peptide could be cleaved by MMPs and penetrate the MMP-overexpressing tumor cells, while in HBE cell line, such MMPs activities would be too low to conduct this enzyme cleavage, leading to no polycation peptide penetration. On the other hand, the MMPs-overexpressed cell lines treated with MMPs inhibitor doxycycline should have lower ACPP cell penetration because MMPs inhibitor doxycycline can decrease the MMPs level.

**Figure 3:**
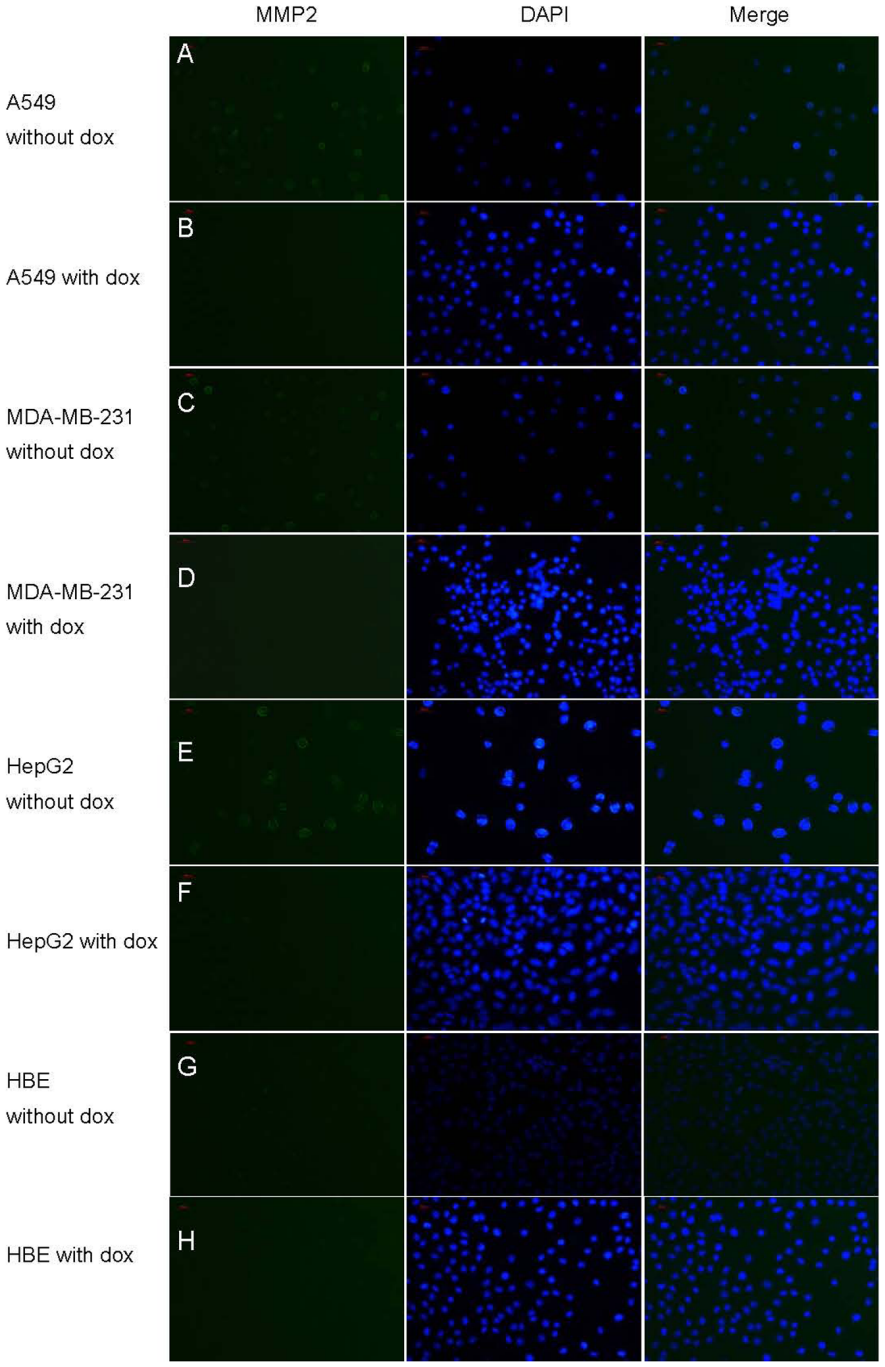
Immunofluorescence analysis of MMP-2 expression in vitro. Immunofluorescence staining for MMP-2 (green) in cytoplasm and nucleus (blue) and merged image (blue-green) in A549, HepG2, MDA-MB-231 and HBE cells SP×200.A, C, E, G show A549, HepG2, MDA-MB-231 and HBE cells receiving no doxycycline treatment. B,D,F,H show the immunofluorescence images of MMP-2 in HepG2, HBE, A549, and MDA-MB-231 cells receiving doxycycline. Scale bar represents 100 px.

**Figure 4:**
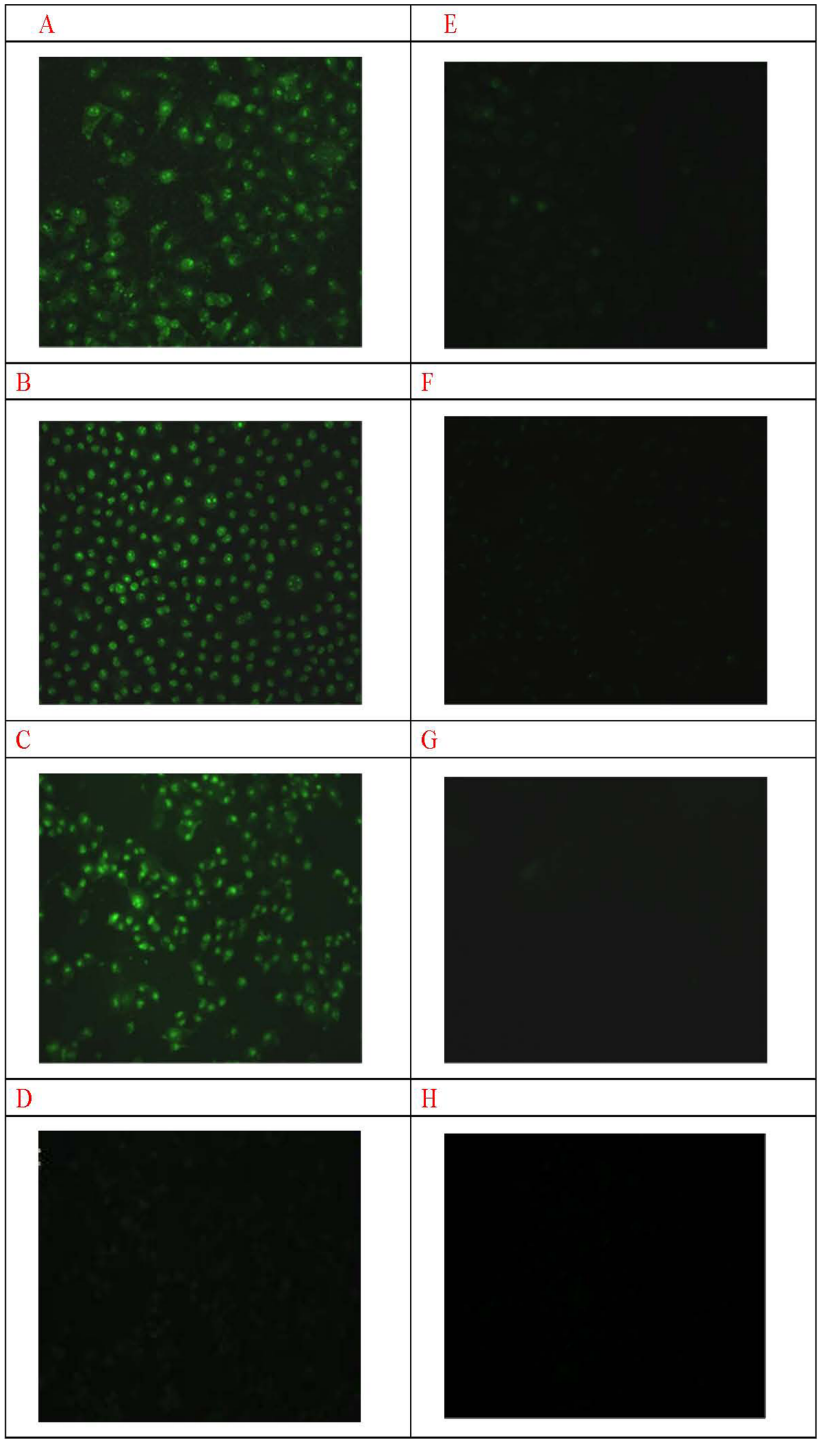
FITC fluorescence of ACPP in A549, MDA-MB-231, HepG2 and HBE. (A-C) A549, MDA-MB-231, HepG2 showed high FITC fluorescence of ACPP SP×200. (D) HBE showed low FITC fluorescence of ACPP SP×200. (E-H) FITC fluorescence of ACPP in A549, MDA-MB-231, HepG2 and HBE treated with Doxycycline SP×200.

The immunofluorescence and ACPP penetration assay were performed to confirm this hypothesis. As shown in Figure 3, immunofluorescence staining displayed the high fluorescence intensity in A549, MDA-MB-231, HepG2 cells and light fluorescence intensity in HBE cell for MMP-2. After the cells were treated with doxycycline for 48 hours, the representative images of immunofluorescence staining in A549, MDA-MB-231, HepG2 cells showed that the immune-activity of MMP2 was dramatically reduced. In Figure 4, the FITC fluorescence of ACPP was strong in A549, MDA-MB-231, HepG2(Figure 4. a-c) while only weak FITC fluorescence could be observed in HBE cells(Figure 4. d).After the doxycycline treatment, the FITC fluorescence of ACPP decreased in A549,MDA-MB-231 and HepG2 cells(Figure 4. e-g), while there was no change on the FITC fluorescence of ACPP in HBE cells(Figure 4. h).

**Figure 5:**
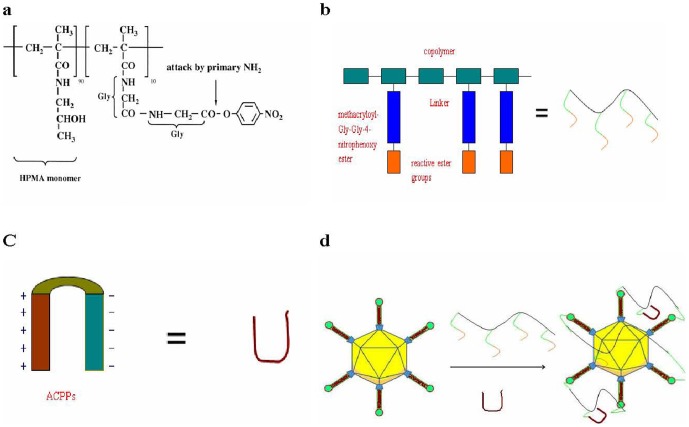
Scheme 1. HPMA copolymers and ACPPs used for adenovirus polymer coating. (a) structure of pHPMA-ONp; scheme for virus modification involving (b) HPMA copolymers used for adenovirus polymer coating; (c) ACPPs used for targeting MMP-overexpressing tumor cells; (d) HPMA copolymers interact with adenovirus and ACPPs, forming a MMP-overexpressing tumor cells targeted polymer-coated adenovirus.

**Figure 6:**
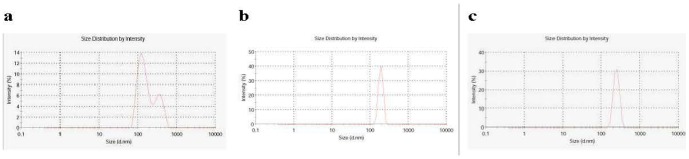
The average hydrodynamic diameters of Ad-eGFP, pc-Ad-eGFP and ACPP- pc–Ad -eGFP conjugates determined by dynamic light scattering (DLS): (a) the average size was found to be 141.8 nm for Ad-eGFP, (b) 189.4 nm for pc-Ad-eGFP, and (c) 236.4 nm for ACPP-pc-Ad-eGFP.

The structure of the random copolymer (pHPMA-ONp) is shown in Scheme 1a in Figure 5. ACPP-pc-Ad-eGFP particles were synthesized and characterized. Polymer-coated Ad5 was prepared by mixing HPMA copolymer (Scheme 1b in Figure 5) with Ad5 and ACPPs (Scheme 1c in Figure 5) in 10% glycerol/PBS (pH 7.8), resulting in polymer molecules linked to each virus particle and ACPPs (Scheme 1d in Figure 5). FITC-labeled ACPPs were included in HPMA copolymers to assay the internalization of ACPPs. Analysis of particle size by dynamic light scattering (DLS) revealed that the average diameter of the unmodified virus was 141.8 nm (Figure 6a), compared with 189.4 nm for the pc-Ad-eGFP particles (Figure 6b) and 236.4 nm for the ACPP-pc-Ad-eGFP particles (Figure 6c).

**Figure 7:**
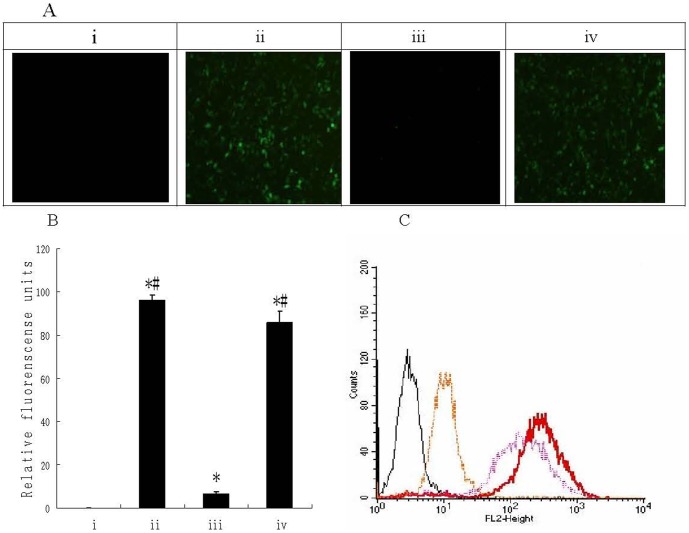
Transduction efficiency of Ad-eGFP, pc-Ad-eGFP and ACPP-pc-Ad-eGFP with A549 cells. (A) A549 cells were seeded into 96-well plates (10^4^ cells/well) and infected after 24 h incubation with 10^4^ particles per cell of Ad-eGFP, pc-Ad-eGFP and ACPP-pc-Ad-eGFP in DMEM/10% fetal calf serum (FCS). Cellular GFP fluorescence was visualized 48 h post-infection using a Nikon TI-S microscope and photographed with a Nikon camera. (i) Uninfected cells; infection with (ii) Ad-eGFP, (iii) pc-Ad-eGFP, and (iv) ACPP-pc-Ad-eGFP. (B) After A549 cells were infected for 48 h as described above, the medium was removed, the cells lysed with 100 ml Triton X-100 (0.2% in H_2_O) and GFP fluorescence was measured (λ_ex_ 488 nm and λ_em_ 538 nm) with a Fluoroskan fluorescence plate reader (Multiskan GO, Thermo Scientific). The columns depict the following: (i) uninfected cells, (ii) Ad-eGFP, (iii) pc-Ad-eGFP, and (iv) ACPP-pc-Ad-eGFP. Data are the means ± SEM. *P<0.05 compared with i, ^#^P<0.05 compared with iii (C) A549 cells were trypsinized, aliquoted at (2×10^5^ cells)/(2 ml DMEM/10% FCS) and incubated in 6-well plates at 37°C until 90% confluence was reached; subsequently, 10^9^ particles Ad-eGFP, pc-Ad-eGFP or ACPP-pc-Ad-eGFP labeled with PI were added. Cells were trypsinized, centrifuged (2 min, 1500 g) and washed in PBS 48 h later. Association of PI-labeled virus with cells was measured using a Coulter EPICS XL flow cytometer with an argon laser (λ_ex_ 540 nm and λ_em_ 625 nm). The fluorescence profile of control cells (black line) or cells infected with virus (red line), pc-virus (yellow line) or ACPP-pc-virus (purple line).

To test the ability of polymer-coated adenovirus to infect cells, the expression of green fluorescent protein (GFP), encoded by the recombinant Ad5 virus (Ad-eGFP), was assayed (Figure 7). When A549 human lung carcinoma cells were infected with 10^4^ particles Ad-eGFP/cell, pc-Ad-eGFP displayed no detectable fluorescent signal at 24 h, while over 90% of A549 cells showed bright fluorescence after infection with ACPP-pc-Ad-eGFP (Figure 7A). Next, GFP expression was measured with a fluorescence plate reader (Figure 7B) to quantitatively measure the ability of the virus to infect A549 cells. We found that pc-Ad-eGFP infection was essentially ablated by the coating procedure, while ACPP-pc-Ad-eGFP penetrated the A549 cells successfully, showing little ablation of GFP expression.

To check the ability of polymer-coated virus to enter cells, the ability of ACPPs to mediate uptake of pc viruses into MMPs overexpressing cells was determined by flow cytometry of A549 cells following 4 h incubation at 37°C with RRPMI-1640, Ad-eGFP, pc- Ad-eGFP (PI) and ACPPs -pc- Ad-eGFP (PI).Uptake of pc-Ad-eGFP(PI) by A549 cells during 4 h incubation at 37°C was effectively abolished by the presence of the polymer coat. However, the ACPPs mediated uptake levelof pc-Ad-eGFP(PI) by A549 cells,which is similar to that of the unmodified Ad-eGFP (PI),seemed almost unaffected by the presence of the polymer coat. (Figure 6c), indicating that ACPPs facilitated the internalization of pc-Ad-eGFP.

**Figure 8:**
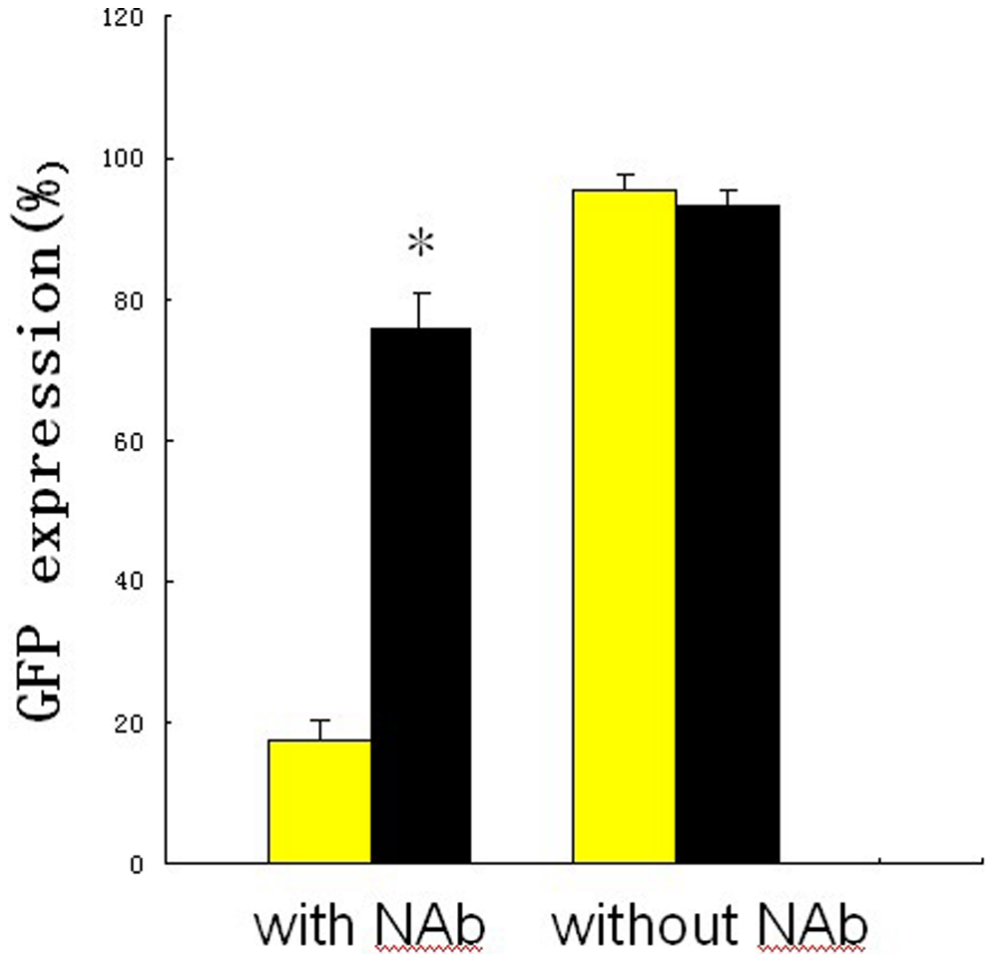
Virus neutralization assay of Ad-eGFP or ACPP-pc-Ad-eGFP. Ad-eGFP or ACPP-pc-Ad-eGFP (10^8^ particles per 100 µl) was incubated with A549 cells in a 96-well plate (10^4^ cells per well; 10^4^ virus particles per cell) in the presence or absence of neutralizing antibodies (NAb). Expression of GFP was measured at 48 h after Triton X-100 lysis and was expressed as a percentage of the fluorescence signal in the absence of human serum. The yellow bar indicates Ad-eGFP, while the black bar denotes ACPP-pc-Ad-eGFP. Data are the means ± SEM. *P<0.05 compared with Ad-eGFP.

The ability of ACPP-pc-Ad-eGFP to evade neutralizing antibodies was tested by examining the inhibition of adenoviral transduction. The modified virus was incubated with human serum known to contain anti-adenovirus neutralizing antibodies, and the polymer coating was found to protect the virus against NAb binding, while NAb significantly inhibited uncoated Ad5 transduction. Figure 8 shows that ACPP-pc-Ad-eGFP is 9-fold more resistant to neutralization than Ad-eGFP. Retargeted pc virus was less susceptible to inhibition of infection than the unmodified virus.

**Figure 9:**
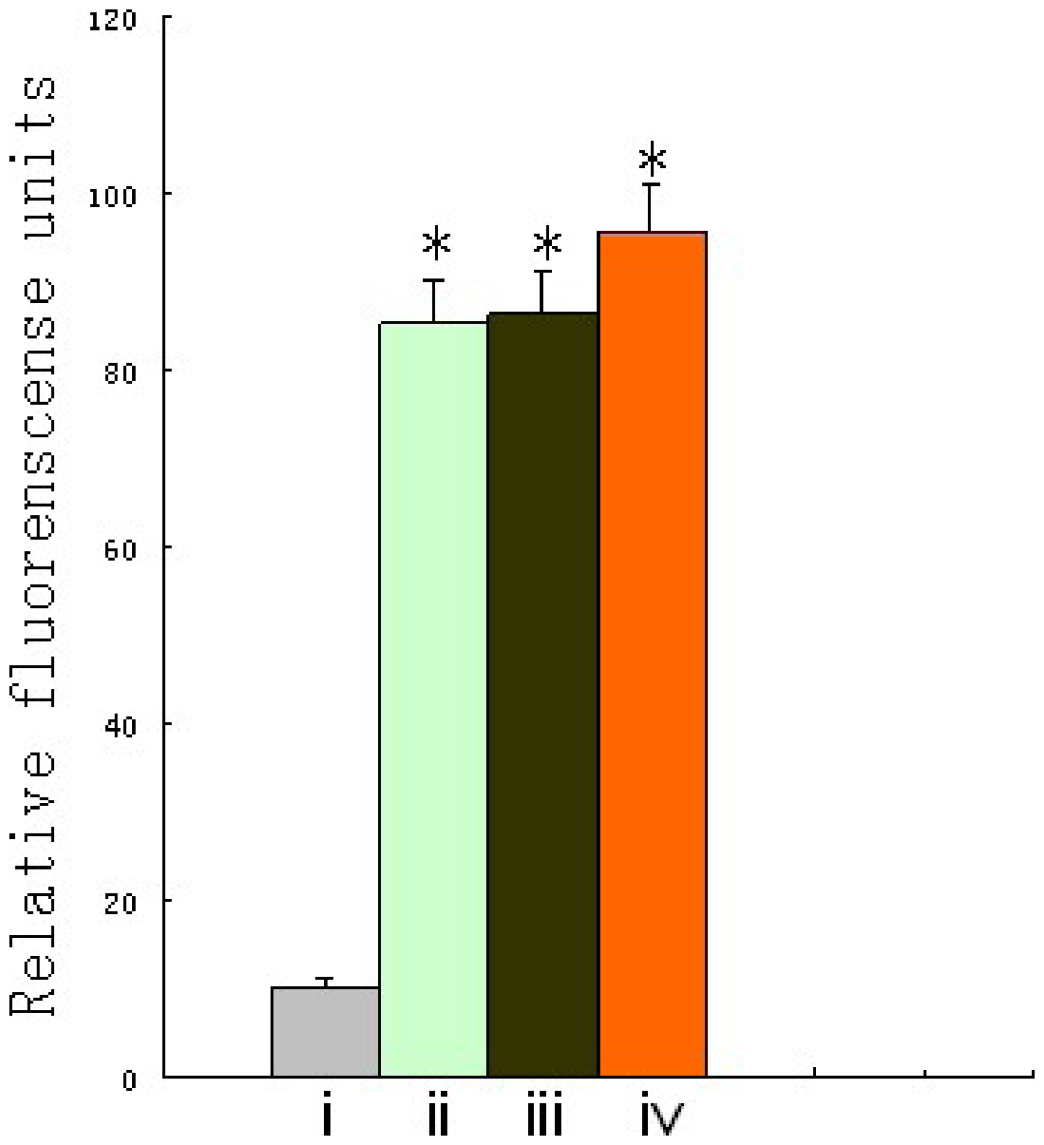
Selective infection of ACPP-pc-Ad-eGFP in MMP-overexpressing cells and control. HBE(control), A549, MDA-MB-231 and HepG2 cells were seeded into 96-well plates (10^4^ cells per well) and after 24 h were infected with 10^4^ particles per cell of ACPP-pc-Ad-eGFP in DMEM/10% FCS (A549, MDA-MB-231) or RRPMI-1640/10% FCS (HBE, HepG2). The supernatant was removed 4 h after infection and incubated with 200 µl DMEM/10% FCS (A549, MDA-MB-231) or RRPMI-1640/10% FCS (HBE, HepG2) for an additional 48 h before fluorescence was measured. The columns depict the following: i, HBE; ii, A549; iii, MDA-MB-231; and iv, HepG2. Data are the means ± SEM. *P<0.05 compared with the HBE cell.

To test the feasibility of retargeting, ACPPs were chosen because these molecules had previously been shown to retarget cargoes to MMP-overexpressing cells. Targeting ACPPs were chemically attached to the reactive ester groups remaining on the surface of the pc virus, and this modified virus was purified from free polymer and free targeting agents using S400 spin columns 27-5140-01 as described previously. After HBE, A549, MDA-MB-231 and HepG2 cells were incubated with ACPP-pc-Ad-eGFP(PI) for 1 h at 37°C, the ability of ACPPs to target the MMP-overexpressing cells was determined by measuring GFP expression. Figure 9 shows that when cell lines were infected with this retargeted virus, the trend in cellular uptake of ACPP-pc-Ad-eGFP(PI) was as follows: A549, MDA-MB-231, HepG2 and HBE (ii, iii, iv and i, respectively). ACPP-pc-Ad-eGFP showed relatively low infectivity in HBE cells, while significantly enhanced GFP expression could be seen in A549, MDA-MB-231, and HepG2 cells, suggesting that selective infection in MMP-overexpressing tumor cells should be feasible.

**Figure 10:**
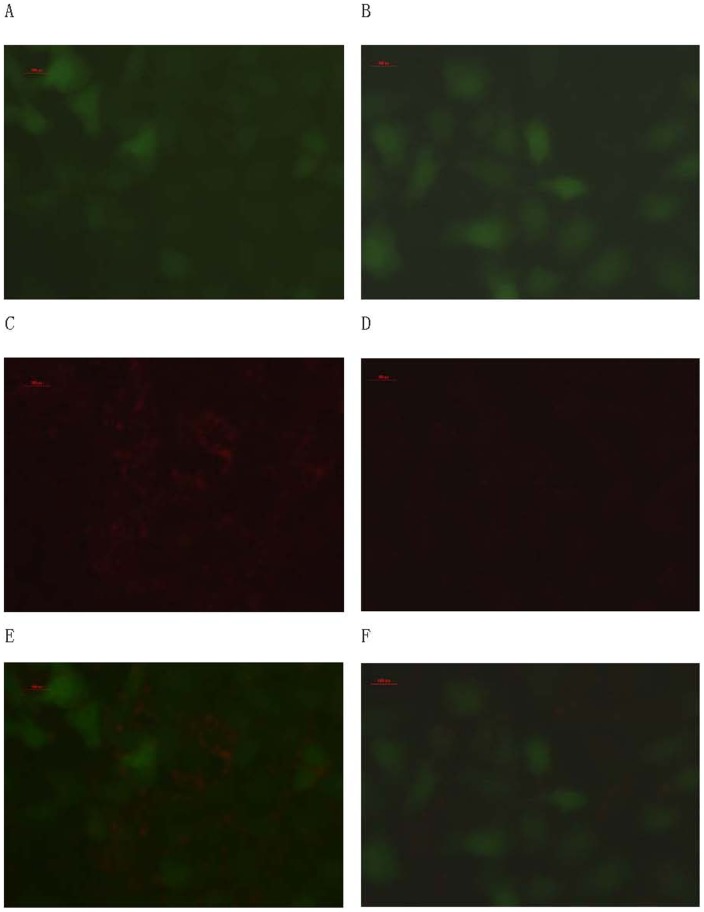
Cytoplasmic delivery of ACPP-pc-Ad-eGFP and pc-Ad-eGFP in A549 cells. Inverted fluorescence microscopy image show(A, B) cytoplasmic marker CFSE (green), (C) incubated ACPP-pc-Ad-eGFP(PI) with A549 cells for 4 h at 37°C yielded uptake of the conjugate (red); (D) incubation pc-Ad-eGFP(PI) with A549 cells for 4 h at 37°C yielded little uptake (red); (E) superimposition of the two dyes (A and C) confirms ACPP-mediated nonendocytotic transport to the cytoplasm. (F) superimposition of the two dyes (Band D) exhibited only endocytotic vesicles. Scale bar represents 100 px.

Cytoplasmic delivery of ACPP-pc-Ad- eGFP was verified with the use of cytoplasm-specific markers. Cytoplasmic import was tested by co-incubating the cells with cytoplasmic marker CFSE (green fluorescence) and ACPP-pc-Ad-eGFP (PI) (red fluorescence). These dyes were found to co-localize resulting in a blue–red signal which demonstrated the cytoplasmic localization of the conjugate. CFSE (Figure 10A and 10B, green) and PI-labelled Ad-eGFP (Figure 10C and 10D, red) were separately visualized by fluorescence microscopy, and superimposition of the two dyes resulted in intracellular blue–red stained areas(Figure 10E and 10F, green–red), confirming the cytoplasmic delivery of ACPP-pc-Ad-eGFP and endocytotic uptake of pc-Ad-eGFP.

**Figure 11:**
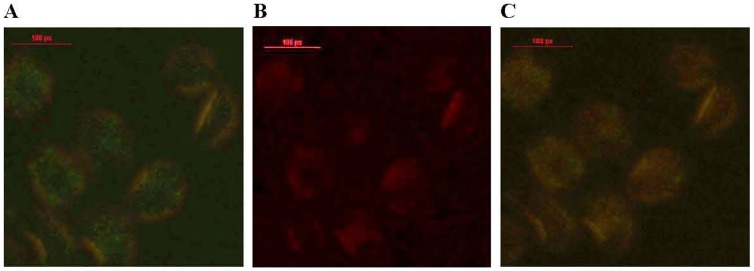
ACPP-pc-Ad-eGFP distributed in cytoplasm of A549 cells. (A) cytoplasmic uptake of ACPP marker FITC (green); (B) cytoplasmic uptake of Ad-eGFP marker PI (red); (C) superimposition of the two dyes (green–red staining) confirms ACPP-mediated transport of pc-Ad-eGFP to the cytoplasm. Scale bar represents 100 px.

The intracellular distribution of the ACPP-pc-Ad-eGFP was studied by fluorescence microscopy. A549 human lung carcinoma cells were incubated with fluorescein-labelled particles [(FITC)ACPP-pc-Ad-eGFP(PI)] for 4 h at 37°C (Figure 11). The ACPP-mediated internalization in the cytoplasm was inferred from diffuse staining visible within the cell body. The FITC on the ACPPs (Figure 11A, green) and the PI on the Ad-eGFP (Figure 11B, red) were separately tracked by fluorescence microscopy. The superimposition of these two dyes resulted in intracellular green-red stained areas (Figure 11C, green-red), which confirmed the cytoplasmic distribution of (FITC) ACPP-pc-Ad-eGFP(PI). These results are significant, as they demonstrate that the intracellular delivery of the conjugate ACPP-pc-Ad-eGFP was mediated by the ACPPs.

**Figure 12:**
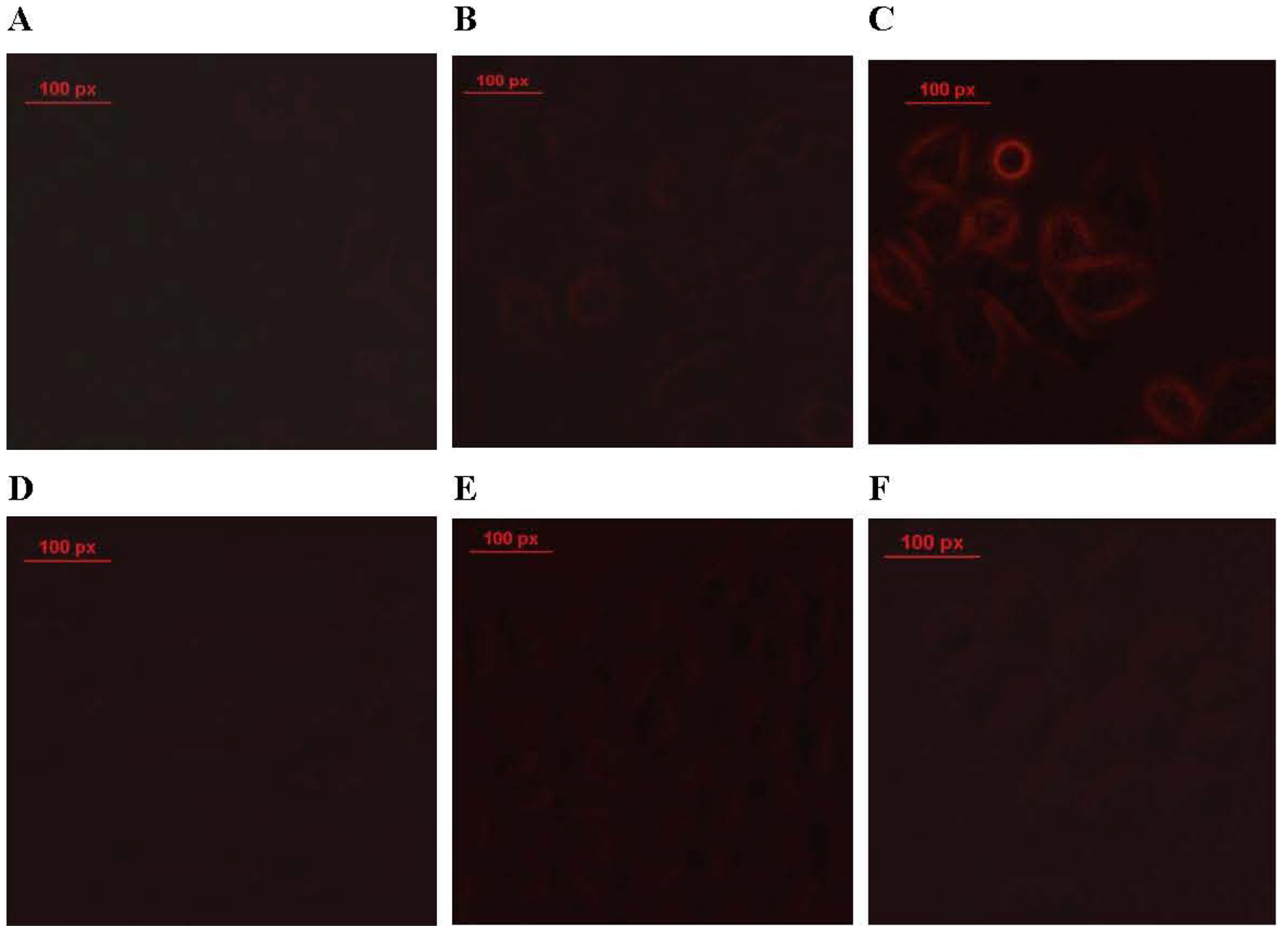
Fluorescence images of A549 cells incubated with ACPP-pc-Ad-eGFP(PI) and pc-Ad-eGFP(PI) after 20 min, 2 h, and 4 h. Panels A-C depict the cytoplasmic uptake of ACPP-pc-Ad-eGFP at 20 min (A), 2 h (B), and 4 h (C). Panels d-f depict the cytoplasmic uptake of pc-Ad-eGFP at 20 min (D), 2 h (E), and 4 h (F). Scale bar represents 100 px.

To investigate the time-dependence of the internalization process, Ad-eGFP was labeled with the fluorescent dye PI and employed in a fluorescence microscopy assay. Studies performed with ACPP-pc-Ad-eGFP(PI) at 37°C showed cytoplasmic uptake of Ad-eGFP(PI) at 20 min, 2 h and 4 h. The intensity of PI fluorescence was significantly stronger (Figure 12A-C) than that of the control pc-Ad-mda-7-eGFP particles lacking an ACPP (Figure 12D-F). Increasing the period of incubation with ACPP-pc-Ad-eGFP resulted in the cells showing a persistent increase in fluorescence intensity within the cytoplasm. The results indicate that the uptake of Ad-eGFP is time-dependent, with localization to the cytoplasm able to occur within 20 min.

## Discussion

The use of ACPPs, which possess a targeting mechanism based on selective local separation from CPPs, is a flexible strategy that concentrates the therapeutic conjugates in the immediate vicinity of MMP-overexpressing solid tumors. By means of proteolytic activation of ACPP, we believe the use of pc-Ad- eGFP conjugates possesses several advantages. First, Uptake of most pc-Ad5 was observed to occur via endocytotic pathway and localized solely in vesicular compartments with no uptake into the cytoplasm,ACPP facilitated the penetration of the pc-Ad-eGFP through the cell membrane directly and the entry into the cytoplasm The localization in the cytoplasm of Ad-eGFP are important for effective expression of therapeutic adenovirus. Second, this system allows the diameter of the delivery cargo to be up to polymers of several nanometers [58]–[60]. As they are appended to the polycationic portion of ACPP, polymers, which often taken up by endocytosis, can be carried into the cytoplasm after linker cleavage. With the ACPPs, the excessive molecular mass overcomes the disadvantage of decreasing penetration into solid tumors with high interstitial fluid pressure. Third, it should be adaptable to a wide variety of solid tumors, as at least 26 members of the MMPs family have been identified, and they are the best characterized proteases overexpressed by tumors [61]–[65].

By modification of the surface of adenovirus with multivalent reactive 4-nitrophenoxy groups, pHPMA ablates their normal receptor binding properties, shields the virus from recognition by neutralizing antibodies, and permits incorporation of target molecules for virus retargeting. The convincing evidence that adenovirus can be targeted to MMP-overexpressing tumors and infect cells efficiently by means of proteolytic activation of cell-penetrating peptides has important implications for the intravenous administration of gene therapy vectors. Our finding that adenoviruses can be retargeted to MMP-overexpressing tumors provides an opportunity to refine their distribution kinetics and their tissue tropism in vivo, and permits the use of lower viral doses by avoiding CAR-mediated infection of non-target tissues. In addition, evidence of diminished neutralization by anti-adenovirus antibodies should enable the use of lower doses. We anticipate that this system will enable successful application of adenovirus gene therapy vectors with significantly decreased toxicity.
